# Spray Drying of Prickly Pear (*Opuntia ficus‐indica* [L.] Mill) Juice: Effects of Different Carriers on Yield and Powder Properties

**DOI:** 10.1111/1750-3841.71221

**Published:** 2026-06-26

**Authors:** Panagiotis Chaloulos, Athina Mourouti, Ioanna Mandala

**Affiliations:** ^1^ Laboratory of Food Process Engineering Department of Food Science & Human Nutrition Agricultural University of Athens Athens Greece

**Keywords:** betalains, *Opuntia*, prickly pear, spray drying

## Abstract

Prickly pear juice (*Opuntia ficus‐indica* [L.] Mill, orange variety) was spray dried with maltodextrin (MD), MD and whey protein isolate in a ratio 199:1 (WPI1), MD and whey protein isolate in a ratio 100:100 (WPI100) (1:1), MD and casein in a ratio 199:1 (Cas) and MD and gum arabic in a ratio 175:25 (GA) (7:1). The ratio of juice solids to carriers was 2:1 in all samples. Large amount of whey protein significantly increased powder yield compared to plain MD (58.47% vs. 43.17%), while the minimal whey protein addition (WPI) exhibited improved yield (49.47%) compared to plain MD. Betalains retention was high in all systems (> 90%) both for betaxanthins and for betacyanins. The retention of betalains during storage for 77 days in the dark was also good in 4°C, 40°C and 60°C, but when the powders were exposed to light in a range of relative humidity (11%–75%), the retention of betaxanthins diminished when the relative humidity exceeded 58%. Storage in relative humidity 44% or higher led to macroscopic powder caking in all systems except for the WPI100 system, in which visible caking appeared in 58% relative humidity. No substantial difference was observed in the absorption curves of the different systems. Understanding the effects and properties of each carrier is crucial when designing appropriate and cost‐effective carrier systems according to the desired properties.

## 1 Introduction

Prickly pear (*Opuntia ficus‐*indica [L.] Mill) is a cactus of Cactacea family which can grow in environments where extreme temperatures and conditions prevail, making it a viable option in areas where other plants cannot survive (FAO 2017). The nutrients of prickly pear, such as vitamin C and antioxidants, give prickly pear medicinal properties beneficial to human health (Rahimi et al. 2019). At the same time, betalains are natural colorants that can yield a wide range of colors (Gandía‐Herrero et al. 2013; García‐Cayuela et al. 2019) so they could be utilized by the food industry. There is also a growing amount of interest in its polysaccharides, namely mucilage, which present both nutritional (Frati et al. 1990) and technological importance (Quinzio et al. 2018; Otálora et al. 2023).

The most common way for drying juices is spray drying, as it can dry the product in a very short time and is a cheaper alternative than freeze drying (Shishir and Chen 2017). However, spray dying of juices rich in sugars can be challenging, as they lead to a sticky powder with poor yields, so the addition of carriers such as maltodextrin, gums or proteins is necessary (Bhandari et al. 1997). Maltodextrin is a very common carrier for spray drying as it is low cost and it has proved to be a successful carrier for betalain encapsulation (Fernández‐López et al. 2018). Whey protein and casein can improve yield even when used in small amounts, as they migrate to the droplet surface after the atomization due to their interfacial properties (Adhikari, Howes, Wood, et al. 2009; Adhikari, Howes, Bhandari, et al. 2009; Jayasundera et al. 2010). This increased yield observed in sucrose solutions has also been confirmed for juices with varying concentrations of sucrose, glucose, and fructose, such as blueberry juice (Fang and Bhandari 2012) and strawberry puree (Gong et al. 2018). However, reports on solutions containing mainly fructose and glucose are scarce (Shi et al. 2013). Several studies on spray drying prickly pear juice have focused on yield. Carmona et al. (2021) have reported the impact of increasing amount of carriers (maltodextrin and cactus cladode mucilage) on yield, while Obón et al. (2009) studied the effect of °Brix and carrier: juice solids ratio on yield. Other studies have included proteins in the carrier system such as gelatin (Castro‐Muñoz et al. 2015), whey protein (Toledo‐Madrid et al. 2018), and soy protein (Robert et al. 2015) but recovery yield is rarely discussed.

The choice of carriers can greatly affect yield and quality of the powders, as well as the protection of sensitive compounds such as betalains both during the exposure at the high temperatures of spray drying and during storage (Robert et al. 2015; Saénz et al. 2009). The stability of betalains in spray dried prickly pear has been fragmentally reported by many studies with varying results depending on relative humidity, temperature, and exposure to light (Fernández‐López et al. 2018; Otálora et al. 2018; Saénz et al. 2009; Robert et al. 2015; Vergara et al. 2014), although it has not been screened to a full extent. Betalains retention is influenced by many factors such as storage temperature, relative humidity and the presence of light (Herbach et al. 2006). Therefore, selection of appropriate carrier system is crucial for the successful spray drying and commercial utilization of prickly pear powder from the food industry. Superior protection is offered when composite matrices are used as wall materials in terms of betalains encapsulation using agri‐food waste. However, there are still challenges to produce stable powders on an industrial scale (Martins et al. 2024; Ha et al. 2024).

To the best of our knowledge the literature data about prickly pear juice encapsulation with various carriers are scarce. Different carriers could enhance received powder stability, as mentioned before. Moreover, prickly pear powders could have multiple uses in different fields of application, enhancing their added value.

The aim of the present study is to investigate how protein addition even at minimal quantities affects powder yield, betalain retention, and storage stability of spray dried prickly pear juice. In addition, the behavior of the spray dried prickly pear powder in a broader range of storage conditions is documented.

## 2 Material and Methods

### 2.1 Materials

For the preparation of juice, prickly pears (*O. ficus‐indica* [L.] Mill) orange variety from Heraklion, Crete (35°19′36″ N 25°08′36″ E) were used. The fruits were harvested in September 2019. They were peeled and frozen at −40°C. Then, they were thawed and their juice was squeezed into tulle to obtain clear juice without seeds and pulp. The juice was kept at −40^ο^C until used. Whey protein isolate (Lacprodan 9224, Arla, Denmark), casein sodium salt from bovine milk (CAS; Sigma Aldrich—C8654), arabic gum (9000‐01‐5, ICN Biomedicals Inc.) and maltodextrin (DE 12–16) were used as carriers for spray drying. All reagents used were of analytical grade.

### 2.2 Spray Drying Process

Preliminary experiments were performed with various solutions (model solution with 10% w/w sucrose, model solution with 5% w/w fructose and 5% w/w glucose, as well as our prickly pear juice). Powder yield and water activity of the powders were assessed to find optimal conditions, in terms of carrier concentration and spray drying parameters (inlet drying temperature, atomizing pressure, and liquid feed rate). After the initial screening, six encapsulation carrier systems were used to conduct the experiment. Each test was performed with 100 g of prickly pear juice, in which 20 g of carriers had been dissolved to maintain a constant ratio of juice solids to encapsulation carriers. The ratios of the encapsulation carriers for each test are shown in Table 1. The ratio of juice solids to carriers was set to 1:2 because it was found to be the least amount of carriers to systematically offer sufficient powder yield for the fructose/glucose solution and prickly pear juice. This ratio actually falls in the range of many previous studies on spray drying of prickly pear juice (Saénz et al. 2009; Robert et al. 2015; Obón et al. 2009; Carmona et al. 2021). The minimal amount of protein is some systems was chosen because it has been shown to greatly improve yield in spray drying of sugar rich materials (Adhikari, Howes, Wood, et al. 2009; Fang and Bhandari 2012). A total of 18 tests were performed, 3 for each carrier system, in random order. The resultant solutions were fed to a spray dryer (SSPC, Edibon, Spain). The spray‐dryer was operated at inlet air temperature of 150°C, atomizing air pressure of 1 bar and a liquid feed rate of 7.5 mL/min. The air flow was 38 m^3^/h. The spray dryer was preheated while using deionized water as a liquid feed to reach equilibrium (approximately 15 min). The resulting outlet temperature during the spray drying of water was approximately 74°C at equilibrium. Then the prickly pear juice solution was fed. The outlet temperature during the spray drying of the juice slightly increased to 77°C, since the samples contained some solids, hence there was less water to be evaporated. No difference was observed among the different samples, since all of them had the same solids concentration. At the end of the procedure, 20 mL deionized water was used to rinse the majority of the juice residue from the feeding beaker, as well as the residue from the pipes of the peristaltic pump. Only the powder gathered at the collector was collected. No other part (e.g., cyclonic separator) was scratched to retrieve additional powder. The powders obtained were stored at −40°C, protected from light. The spray dryer can be seen in Figure S1.

**TABLE 1:** Ratios of carriers and soluble solids of prickly pear juice.

Carrier System	Sample name
Juice solids:maltodextrin, 100:200	MD
Juice solids:matlodextrin:whey protein isolate, 100:199:1	WPI 1
Juice solids:matlodextrin:whey protein isolate, 100:100:100	WPI 100
Juice solids:matlodextrin:gum arabic, 100:175:25	GA
Juice solids:matlodextrin:casein, 100:199:1	Cas

#### 2.2.1 Powder Yield

The powder yield was calculated from the formula:

$$\mathrm{Powder}\;\mathrm{yield}\;\;\left(\%\right)=\frac{w_{p}}{w_{s}}\;\times 100$$

where *w*_p_ is the weight of the dust collected in the collector and *w*_s_ is the weight of the solids present in the original feed liquid (30.2 g).

### 2.3 Juice and Powder Analysis

#### 2.3.1 Betalains Analysis

The betalains analysis were performed spectrophotometrically according to the method of Stintzing et al. (2005). First, a quantity of juice was filtered through a Whatman nylon syringe filter (0.20 µm). Then, either the filtrate (for juice analysis) or the dried prickly pear powder (for the powder analysis) was diluted with McIlvaine buffer (pH 6.5) to obtain absorbance values of 0.1–1.0. Betacyanins pigments were monitored at 538 nm, and betaxanthins were monitored at 480 nm. The content of betacyanins or betaxanthins in the juice was determined according to the Lambert—Beer equation:

$$\mathrm{Betacyanin}/\mathrm{betaxanthin}\;(\mathrm{mg}/L)\;=\;\frac{A\times \mathrm{DF}\times \mathrm{MW}\times 1000}{\varepsilon \times L}$$

where *A* is the absorption, DF is the dilution factor, MW the molecular weight, *ε* the molecular extinction coefficient, and *L* is the path length of the cuvette (1 cm). For the quantification of betacyanins the molecular weight (MW) and molecular extinction coefficients (*ε*) of betanin were used (MW_538_ = 550 g × mol^−1^ and *ε*_538_ = 60,000 L × mol^−1^ × cm^−1^) while for the betaxanthin content indicaxanthin was used as a reference (MW_480_ = 308 g × mol^−1^ and *ε*_480_ = 48,000 L × mol^−1^ × cm^−1^).

#### 2.3.2 Stability Studies of Encapsulated Pigments

Powders were weighed (approximately 0.1 g of each sample, weighted and recorded precisely in an analytical balance) in test tubes and stored in absence of light, at 4°C, 40°C, and 60°C. Taking the initial concentration as reference of time zero, the samples were withdrawn every 7, 14, 28, and 77 days for the measurement of betalain content. Also, powders were weighed (0.2 g of sample) and stored at different humidities (0.11–0.75) for 77 days in the presence of light. Supersaturated salt solutions (LiCl, MgCl_2_, K_2_CO_3_, NaBr, NaCl) were used to create the different relative humidity conditions.

Τhe powders were diluted with McIlvaine buffer (pH 6.5, citrate‐phosphate) to obtain absorbance values of 0.1–1.0. The content of betacyanins and betaxanthins was determined as described in Section 2.3.1.

#### 2.3.3 Soluble Solids, pH, Acidity, and Vitamin C

Soluble solids (°Brix; WYA 1S ABBE Refractometer), pH (HI98150, Hanna Instruments Inc.), and titratable acidity (based on AOAC 942.15) of the juice were determined. The vitamin C content of the juice was determined via titration with 2,6‐dichlorophenylindophenol (DPIP) as indicator. The sample was diluted with metaphosphate–acetic acid solution (3% w/v and 8% vol, respectively) and titrated with a standard DPIP solution, based on the AOAC 967.21 method (AOAC 2019).

#### 2.3.4 Water Content and Water Activity

Water content was determined by drying 5 g of powder in a vacuum oven (approx. 60 Torr) at 70°C to constant weight. The procedure followed was based on the AOAC 22.013 method (AOAC 2019). The determination of water activity (*a*_w_) was performed at 25°C using the Hygrolab C1 instrument (Rotronic AG, Switzerland).

#### 2.3.5 Tap Density

Tap density (g/mL) was determined by measuring the volume occupied by approximately 1 g of powder in a 10 mL volumetric tube after stirring in the vortex for 1 min based on previous study (Chaloulos et al. 2021). The samples were analyzed in triplicate and the means were recorded. Tap density (*ρ*) was calculated as follows:

$$\rho =\frac{m}{V}$$

where *m* is the weight of the powder, *V* is the volume of the powder after 1 min stirring.

#### 2.3.6 Color Measurement

The CIE Lab parameters (L*, a*, b*, C*, h°) of encapsulates were measured by using a 3nh High‐Quality Spectrophotometer NS800S colorimeter (Shenzhen 3NH Technology CO., LTD., Color controller HK Limited, Xili, NanshanDistrict, Shenzhen, China) based on previous study (Chaloulos et al. 2021).

#### 2.3.7 Sorption Isotherms

The moisture absorption isotherms of the spray dried powders were determined by the static gravimetric method at 25°C, similarly to previous studies (I. Mandala et al. 2009) using supersaturated salt solutions (LiCl, MgCl_2_, K_2_CO_3_, NaBr, and NaCl with water activities of 0.11, 0.33, 0.43, 0.58, and 0.75, respectively). The powders were weighed (approximately 0.2 g) in vials and were placed in a desiccator with SiO_2_ for 2 weeks. This step is needed because all samples must be in absorption phase, since absorption and desorption often yield different moisture levels for the same water activity. Our samples initial water activity was above 0.11, which was the lowest water activity used (LiCl), therefore SiO_2_ placement ensured that all samples dehydrate to a water activity level below 0.11, so that all samples will later equilibrate to their absorption phase in every salt solution. Then, they were transferred to a jar containing the respective salt solution. In the saline solutions with the highest water activity (NaCl), an open vial containing thymol was added to prevent fungal growth. The moisture adsorption isotherm data were modeled according to Guggenheim, Anderson, and de Boer (GAB) model:

$$\;x_{\mathrm{eq}}=\frac{x_{m}\times C\times k\times a_{w}}{\left(1-k\times a_{w}\right)\times \left(1+\left(C-1\right)\times k\times a_{w}\right)}$$

where *x*_eq_ is the water content at equilibrium, *x*_m_ is the monolayer water content, while *C* and *k* are dimensionless parameters of the GAB model.

Coefficient of determination (*R* ^2^) was evaluated, as well as the relative mean error:

$$E=\frac{100}{N}\;\sum_{i=1}^{N}\frac{\left|m_{i}-m_{p}\right|}{m_{i}}$$

where *m*_i_ are the experimental values, *m*_p_ are the predicted values, and *N* is the population of the experimental data.

Only GAB model is presented because its fit was equal or better compared to other models tested (Brunauer, Emmett, and Teller model and Peleg model). GAB model is able to adapt to a large range of water activity, while also having a physical meaning (Kaderides and Goula 2017).

### 2.4 Statistical Analysis

All measurements were made in triplicate. Statistical processing was performed with Statgraphics Centurion XVI (Statgraphics Technologies, Inc., The Plains, Virginia).

The Fisher LSD method was used to determine the significant differences between samples (*p*‐value < 0.05).

## 3 Results and Discussion

### 3.1 Characteristics and Bioactive Components of the Juice

Τhe physical and chemical properties of the juice used in the present study are summarized in Table 2. Total soluble solids appear to be at lower levels than similar studies in orange variety of *O. ficus‐indica* (L.) Mill (Stintzing et al. 2005; Carmona et al. 2021; Fernández‐López et al. 2018). Betaxanthins (yellow pigments) were more abundant than betacyanins (red). More specifically, the yellow pigments amounted to 13.57 mg of betaxanthin/100 mL of juice while the red ones to 1.67 mg of betacyanin/100 g of juice. These results are within the range of previous studies on orange varieties of *O. ficus‐indica* (L.) Mill (Stintzing et al. 2005; Carmona et al. 2021; Fernández‐López et al. 2018; Morales et al. 2009).

**TABLE 2:** Physical and chemical characteristics of orange prickly pear juice.

Parameters	Value
Moisture (% w.b.)	89.88 ± 0.04
pH	5.88
Acidity (citric acid% w/w)	0.039 ± 0.001
Total soluble solids (°Brix)	10.2 ± 0.1
Vitamin C (mg/100 mL juice)	9.82 ± 1.09
Betaxanthins (mg indicaxanthin/100 mL juice)	13.6 ± 0.1
Betacyanins (mg betanin/100 mL juice)	1.7 ± 0.1

### 3.2 Powder Yield

As shown in Table 3, the powder yields range between 41.20% and 58.47%. The highest values were observed in the samples that used whey protein in the carrier system, with the WPI100 system exhibiting substantially higher yield (58.47%) reaching statistical significance compared to all the other systems, despite the high standard deviation. The WPI1 system also had a high yield (49.47%), reaching statistical significance only compared to GA (41.20%). Previous studies on spray drying of sucrose solutions with either whey protein or casein have reported the beneficial role of the proteins regarding powder yield (Adhikari, Howes, Bhandari, et al. 2009; Adhikari, Howes, Wood, et al. 2009; Fang et al. 2013; Jayasundera et al. 2010). It has been suggested that the proteins migrate to the droplet surface almost immediately after the atomization due to their interfacial properties (Jayasundera et al. 2010; Nijdam et al. 2014; Drusch et al. 2012). The ratio of the protein on the particle surface exceeds 50% even if the ratio of the protein in the bulk is as low as 0.25%–0.50% (Fang et al. 2013; Fang and Bhandari 2012). The higher protein ratio of the surface leads to an increased glass transition temperature (*T*_g_) for the particle surface compared to the *T*_g_ of the bulk, given that the protein has a higher *T*_g_ compared to sucrose, fructose, and glucose, which results in a less sticky surface (Bhandari et al. 1997; Adhikari, Howes, Bhandari et al. 2009; Jayasundera et al. 2010). It has also been reported that using surfactants to displace the proteins from the droplet surface leads to a substantial decrease in the powder yield (Jayasundera et al. 2010). In our study, the juice contains mainly glucose and fructose, which have even lower *T*_g_ compared to sucrose (Bhandari et al. 1997). Preliminary trials with aqueous solutions of the same °Brix with either plain sucrose or mix of glucose and fructose (1:1) were carried out. In our drying setup sucrose behaved very well both with whey protein and casein, yielding powder recoveries > 60% at solids ratio of 99:1 (sucrose:protein). However, these yields plummeted (0%) when a mixture of fructose:glucose (1:1) was used with the same sugars to protein ratio (99:1). The same was observed for the prickly pear juice (juice solids:protein = 99:1). After more trials of increasing maltodextrin, the ratio of 100:200 (juice solids:maltodextrin) was chosen in order to get sufficient yield, since below a ratio of 100:150 (juice solids:maltodextrin), the powder recovery was marginal. The concentration of MD is crucial, since at a high ratio of total juice solids:MD (1:1) the glass transition temperature increases exponentially, independently of the sugar composition of the juice (Grajales‐Lagunes et al. 2025).

**TABLE 3:** Effect of different carrier systems on the properties of spray dried prickly pear juice.

	MD	WPI 1	WPI 100	GA	Cas
Yield (%)	43.17 ± 1.61^ab^	49.47 ± 4.94^b^	58.47 ± 3.54^c^	41.21 ± 2.86^a^	45.10 ± 6.76^ab^
Water content (%w.b.)	4.89 ± 0.94^a^	5.05 ± 0.65^a^	4.77 ± 0.75^a^	5.29 ± 0.43^a^	4.77 ± 0.73^a^
Water activity	0.261 ± 0.016^abc^	0.276 ± 0.012^bc^	0.248 ± 0.019^a^	0.279 ± 0.024^c^	0.272 ± 0.006^abc^
Tap Density (g/mL)	0.35 ± 0.03^a^	0.37 ± 0.03^a^	0.35 ± 0.01^a^	0.36 ± 0.02^a^	0.38 ± 0.04^a^
Preservation of betaxanthins (%)	94	94	96	94	91
Preservation of betacyanins (%)	110	112	141	114	111

*Note*: Values are expressed as mean ± standard deviation. The averages followed by the same letter on the same line do not differ significantly (*p* > 0.05).

### 3.3 Powder Characteristics

#### 3.3.1 Water Content and Water Activity

All powders have *a*_w_ values lower than 0.3, similarly to those reported by Castro‐Muñoz et al. (2015) in their study of spray‐dried prickly pear juice with maltodextrin and gelatin carriers in different ratios. These low values of water activity allow us to consider that these powders are biochemically and microbiologically stable, extending their shelf life. Water content in the final powders remained between 4.77% and 5.30% w.b., with no significant difference.

#### 3.3.2 Tap Density

Tap and bulk density is an important economic factor as it affects the packaging and transport costs of the product (Mandala and Protonotariou 2021). According to Bae and Lee (2008), increasing the MD ratio resulted in higher bulk density, possibly due to a more compact physical structure and a hydrophilic wall matrix. In our study the tap density ranges from 0.35 to 0.38 g/mL; however, there was not any correlation with the ratio of carriers to maltodextrin.

#### 3.3.3 Powder Color

The results from the color measurement of the powders are shown in Table 4. The L* parameter, which represents brightness, was significantly lower (*p* < 0.05) for the WPI100 system, which also exhibited a lower a* value. The betalain content did not differ significantly among the different carrier systems after drying, as it will be discussed further below. Therefore, the difference in color is attributed to the different composition of the particle surface. Adhikari, Howes, Bhandari, et al. (2009) observed that by adding 0.5% whey protein or casein to a sucrose solution, more than 50% of the particle surface was composed of protein. Therefore, it is possible that the lighter color observed in the powders encapsulated with the WPI100 system is due to the strong presence of the protein on the surface of the powders which prevents the appearance of the color of the pigments found in the core of the particles. However, it has to be noted that according to the aforementioned study, one would expect a similar trend for the WPI1 and Cas systems, contrary to our findings. As discussed in the yield section, many studies have demonstrated that the occupation of the particle surface by these proteins approaches saturation in very low ratios of protein in the bulk (Adhikari, Howes, Bhandari, et al. 2009; Adhikari, Howes, Wood, et al. 2009). However, these observations have been made in pure solutions containing only sucrose and protein. The presence of other surfactants can displace the proteins from the surface (Adhikari, Howes, Wood, et al. 2009). In the aforementioned study, the protein content of the particle surface was similar (50%–55%) whether the protein content of the bulk was 0.5% or 1%. However, when a surfactant was added, the protein content of the surface plummeted, with the decrease being more potent for the 0.5% compared to the 1% protein concentration on the bulk (9% and 23% protein on the particle surface, respectively). Therefore, it is possible that the low ratio of protein we used in WPI1 and Cas systems may not be high enough to cause saturation of protein on the particle surface when more complex mixtures such as fruit juices are concerned. The presence of dietary fibers like prickly pear mucilage could also affect the lowest protein ratio in the bulk where protein content of the particle surface approaches saturation.

**TABLE 4:** The color of orange prickly pear powder as described by means of the parameters L*, a*, b*, C*, and *h*°.

	MD	WPI 1	WPI 100	GA	Cas
L*	79.40 ± 0.11^a^	80.22 ± 0.03^a^	82.77 ± 0.32^b^	79.23 ± 0.50^a^	79.89 ± 0.09^a^
a*	15.38 ± 0.07^b^	15.20 ± 0.36^b^	11.93 ± 0.74^a^	15.30 ± 0.66^b^	14.80 ± 0.62^b^
b*	10.46 ± 0.21^bc^	10.60 ± 0.30^bc^	9.32 ± 0.07^a^	11.03 ± 0.65^c^	9.89 ± 0.74^ab^
C*	18.60 ± 0.17^b^	18.55 ± 0.48^b^	15.15 ± 0.55^a^	18.86 ± 0.90^b^	17.80 ± 0.93^b^
*h°*	34.20 ± 0.46^ab^	34.90 ± 0.53^ab^	38.02 ± 1.94^c^	35.79 ± 0.69^b^	33.73 ± 0.94^a^

*Note*: Values are expressed as mean ± standard deviation. The averages followed by the same lowercase letter on the same line do not differ significantly (*p* > 0.05).

#### 3.3.4 Betalains Preservation after Drying

The betalains content of the dried powders is shown in the second column (Day 0) of Tables 5 and 6 for betaxanthins and betacyanins, respectively. No significant difference was observed among the different carrier systems for betaxanthins. However, WPI100 exhibited significantly higher betacyanins content. Expressing these results as a preservation percentage of each betalain group (Table 3) reveals that the betacyanins exceeds 100% for all samples, especially for WPI100 (141%). This means that the betacyanins in the dried powder are more than those in the initial prickly pear solution that was fed into the spray dryer. This may be attributed to the degradation of betaxanthins into products that absorb close to the wavelengths of betacyanins. Various structural modifications may take place during thermal treatment of betalains solutions, which may lead to products that absorb in different wavelength or even are colorless (Herbach et al. 2004, 2006). Although the inverse perspective is often considered for beetroot (Herbach et al. 2006), it has to be noted that in our study the initial composition was heavily in favor of betaxanthins, so by‐products of their degradation could lead to a slight increase of the pigments that absorb to the betacyanin area. After all, the increase in betacyanins content of the WPI100 (+41%) corresponds to a small absolute increase (approximately 2.3 mg/100 g), which is a small amount compared to the betaxanhins content (43.2 mg/100 g). The degradation of betalains is affected by many factors such pH and the matrix (Castellar et al. 2003; Khan 2016). Finally, we cannot eliminate the possibility of Maillard reaction contributing to the absorbance of the betalains, due to the increased protein content and the high temperatures of spray drying. Nonetheless, other analytical techniques such as high‐performance liquid chromatography are required to validate or disprove such speculations, but this is outside the scope of the present study. To the best of our knowledge, there is a gap in the deeper understanding of thermal degradation of betaxanthin rich materials such as orange varieties of prickly pear. The retention of betaxanthins after drying is very high in all systems, with a preservation of over 90%. The retention of betaxanthins encapsulated in the WPI 100 system were the highest (96.16%). Betaxanthins from yellow–orange prickly pear (*Mandarina* variety) presented a high stability, higher than that from beetroot (Carreón‐hidalgo et al. 2022). Other studies have also found good retention of betalains after spray drying with similar carrier systems. These results are in line with studies in which maltodextrin has been used for encapsulation of prickly pear betalains (Robert et al. 2015; Saénz et al. 2009). Carmo et al. (2018) reported high retention for betalains after spray drying with either maltodextrin or maltodextrin combined with whey protein isolate (91.6% and 95.7%, respectively).

**TABLE 5:** Concentration of betaxanthin in powders from prickly pear juice stored for 77 days at 4°C, 40°C, and 60°C in the dark (mg betaxanthins/100 g powder).

Carrier system	Day 0	7 days	14 days	28 days	77 days
**4°C**					
MD	42.2 ± 0.6^Ab^	40.9 ± 0.87^ABa^	41.7 ± 0.4^Aab^	40.7 ± 0.9^Aa^	41.4 ± 0.3^Aab^
WPI 1	42.2 ± 1.1^Aa^	41.5 ± 1.38^ABa^	41.5 ± 0.2^Aa^	41.4 ± 0.3^Aa^	41.0 ± 1.1^Aa^
WPI 100	43.2 ± 0.5^Aab^	43.2 ± 1.41^Ba^	42.7 ± 1.5^Aab^	44.1 ± 0.5^Bab^	44.9 ± 0.2^Bb^
GA	42.1 ± 0.7^Aa^	42.5 ± 1.41^ABa^	41.1 ± 2.3^Aa^	40.8 ± 1.4^Aa^	41.6 ± 1.0^Aa^
Cas	40.7 ± 0.9^Aa^	41.7 ± 0.78^ABa^	42.0 ± 2.0^Aa^	40.7 ± 1.2^Aa^	40.3 ± 1.3^Aa^
**40°C**					
MD	42.2 ± 0.6^Aa^	43.3 ± 1.4^BCa^	42.0 ± 2.0^ABa^	41.7 ± 0.1^Aa^	43.7 ± 1.0^Aa^
WPI 1	42.2 ± 1.1^Aa^	42.1 ± 1.0^ABCa^	41.8 ± 1.1^ABa^	42.4 ± 0.1^ABa^	43.4 ± 1.0^Ba^
WPI 100	43.2 ± 0.5^Aa^	43.8 ± 1.1^Ca^	43.7 ± 0.5^Ba^	44.1 ± 0.1^Ca^	46.1 ± 0.2^Bb^
GA	42.1 ± 0.7^Aab^	40.9 ± 2.3^ABa^	41.5 ± 0.6^ABa^	44.7 ± 0.7^Cc^	44.1 ± 1.0^Abc^
Cas	40.7 ± 0.9^Aa^	41.1 ± 1.9^ABCab^	41.1 ± 2.1^ABab^	42.5 ± 0.4^Bab^	43.5 ± 1.3^Ab^
**60°C**					
MD	42.2 ± 0.6^Aa^	42.59 ± 2.7^Aa^	42.1 ± 1.1^Aa^	40.4 ± 1.4^Aa^	38.7 ± 2.2^Aa^
WPI 1	42.2 ± 1.1^Ab^	41.80 ± 1.1^Ab^	41.7 ± 0.2^Ab^	40.4 ± 1.2^Ab^	38.7 ± 2.4^Aa^
WPI 100	43.2 ± 0.5^Aa^	43.43 ± 0.6^Aa^	46.5 ± 1.5^Bb^	45.1 ± 0.7^Bab^	49.4 ± 2.6^Bc^
GA	42.1 ± 0.7^Aa^	41.54 ± 1.0^Aa^	40.9 ± 2.3^Aa^	40.6 ± 1.2^Aa^	39.8 ± 2.6^Aa^
Cas	40.7 ± 0.9^Aa^	41.08 ± 1.6^Aa^	41.1 ± 0.5^Aa^	41.2 ± 1.2^Aa^	37.9 ± 2.0^Ab^

*Note*: Values are expressed as mean ± standard deviation. The averages followed by the same lowercase letter on the same line do not differ significantly (*p* > 0.05) in relation to the storage time. The averages followed by the same uppercase letter in the same column for the same temperature do not differ significantly (*p* > 0.05) in relation to the carrier system used.

**TABLE 6:** Concentration of betacyanin in powders from prickly pear juice stored for 77 days at 4°C, 40°C, and 60°C in the dark (mg betacyanin/100 g powder).

Carrier system	Day 0	7 days	14 days	28 days	77 days
**4°C**					
MD	6.1 ± 0.1^Abc^	6.0 ± 0.1^Ab^	6.3 ± 0.2^Ac^	6.0 ± 0.1^Aab^	5.7 ± 0.2^Aa^
WPI 1	6.2 ± 0.2^Aa^	6.0 ± 0.1^Aa^	6.2 ± 0.1^Aa^	6.0 ± 0.2^Aa^	5.9 ± 0.3^Aa^
WPI 100	7.8 ± 0.5^Ba^	7.5 ± 0.5^Ba^	7.7 ± 0.6^Ba^	7.2 ± 0.6^Ba^	7.5 ± 0.4^Ba^
GA	6.3 ± 0.2^Abc^	6.4 ± 0.3^Ac^	6.6 ± 0.2^Ac^	6.1 ± 0.1^Aab^	5.9 ± 0.1^Aa^
Cas	6.2 ± 0.2^Aa^	6.3 ± 0.4^Aa^	6.2 ± 0.5^Aa^	6.1 ± 0.1^Aa^	6.0 ± 0.3^Aa^
**40°C**					
MD	6.1 ± 0.1^Aa^	6.1 ± 0.1^Aa^	6.2 ± 0.3^Aa^	6.1 ± 0.3^Aa^	6.2 ± 0.2^Aa^
WPI 1	6.2 ± 0.2^Aa^	5.9 ± 0.2^Ab^	6.0 ± 0.1^Aab^	6.2 ± 0.2^Aab^	6.2 ± 0.1^Aa^
WPI 100	7.8 ± 0.5^Ba^	7.2 ± 0.6^Ba^	7.4 ± 0.5^Aa^	7.4 ± 0.8^Aa^	7.9 ± 0.4^Aa^
GA	6.3 ± 0.2^Aab^	5.9 ± 0.4^Aa^	6.2 ± 0.1^Aab^	6.3 ± 0.3^Aab^	6.5 ± 0.1^Ab^
Cas	6.2 ± 0.2^Aa^	6.2 ± 0.8^Aa^	6.3 ± 0.6^Aa^	6.5 ± 0.1^Aa^	6.5 ± 0.7^Aa^
**60°C**					
MD	6.1 ± 0.1^Aa^	6.1 ± 0.1^Aa^	5.7 ± 0.4^Aab^	5.5 ± 0.3^Ab^	5.3 ± 0.1^Ab^
WPI 1	6.2 ± 0.2^Aa^	5.8 ± 0.2^Aab^	5.9 ± 0.5^Aab^	5.5 ± 0.4^Ab^	5.5 ± 0.1^Ab^
WPI 100	7.8 ± 0.5^Bab^	7.3 ± 0.5^Ba^	8.2 ± 0.3^Bab^	8.6 ± 0.7^Bb^	12.6 ± 1.2^Bc^
GA	6.3 ± 0.2^Aab^	6.0 ± 0.2^Aa^	6.1 ± 0.1^Aab^	6.0 ± 0.1^Aab^	5.9 ± 0.1^Ab^
Cas	6.2 ± 0.2^Aa^	6.1 ± 0.6^Aa^	6.0 ± 0.6^Aa^	6.0 ± 0.4^Aa^	5.7 ± 0.4^Aa^

*Note*: Values are expressed as mean ± standard deviation. The averages followed by the same lowercase letter on the same line do not differ significantly (*p* > 0.05) in relation to the storage time. The averages followed by the same uppercase letter in the same column for the same temperature do not differ significantly (*p *> 0.05) in relation to the carrier system used.

#### 3.3.5 Storage Stability of Betalains

The powders collected by spray drying were stored at 4°C, 40°C, and 60°C for 77 days. The betaxanthins and betacyanins content of the different systems is shown at Tables 5 and 6, respectively. A very good retention was observed at all temperatures even after 77 days. No substantial difference is observed with the exception of an increase in betaxanthins and betacyanins after 77 days at 60°C. However, it has to be noted that this temperature is too high even for an accelerated storage so it should be considered only as a rough indication of how stable betalains generally are in the encapsulated systems.

The above results are in line with the observations of Fernández‐López et al. (2018), where betaxanthins were found to be relatively stable at 4°C and 20°C for up to 60 days in the dark, although a substantial decrease was observed at 20°C after the 60 days. Gandía‐Herrero et al. (2013) reported good stability for miraxanthin V (a betaxanthin) and betanidin (a betacyanin) at 20°C in the dark when maltodextrin was used, but poor stability was observed when chitosan was used instead of maltodextrin. Exposure to light also led to rapid deterioration. Finally, Díaz‐Sánchez et al. (2006) also reported high betanin retention (86.2%) in spray dried prickly pear juice encapsulated with maltodextrin after 24 weeks of storage at 25°C in the dark.

**FIGURE 1:**
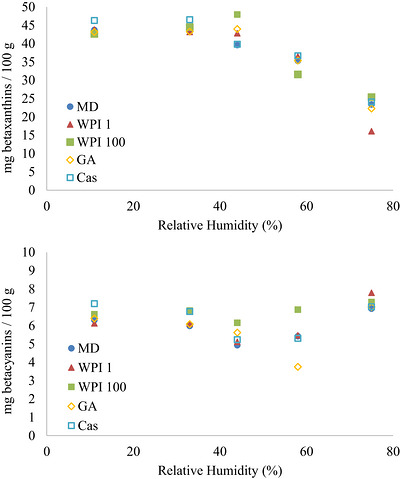
Preservation of betaxanthins and betacyanins in prickly pear juice powders during storage at RH = 11%–75% for 77 days in the presence of light.

Powders were also placed in jars with various relative humidity conditions (11%–75%) in the presence of light for a total period of 77 days. The stability of the betalains under light is shown in the diagrams in Figure 1. Betaxanthins presented good stability in relative humidity up to 44%, despite the exposure to light. However, higher relative humidity substantially decreased the betaxanthin preservation. Otálora et al. (2018) studied the stability of a betaxanthin extract encapsulated with maltodextrin and *O. ficus‐indica* mucilage. They stored the particles in high relative humidity (57% and 90%) in the absence of light for 30 days. They reported a retention rate of approximately 70% for 57% RH and an even lower retention for 90%. However, the carrier ratio in their study was 1:1.25 (juice solids:carriers), which is lower than what we used in the present study (1:2).

#### 3.3.6 Sorption Isotherms

**FIGURE 2:**
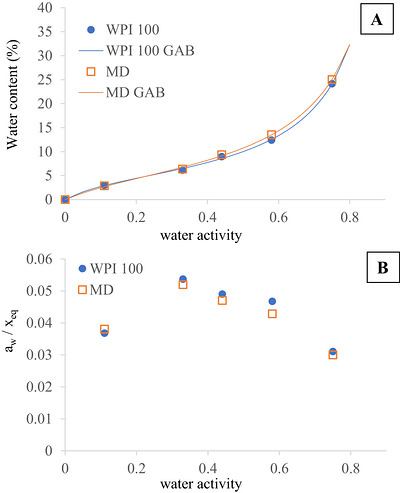
Moisture adsorption isotherms of the spray dried prickly pear juice. (A) Water content plotted against water activity; (B) water activity/water content ratio plotted against water activity.

The moisture absorption isotherms for MD and WPI100 are shown in Figure 2. No big differentiations are observed. All systems exhibited type II isotherm, based on the Brunauer's classification, as a maximum point can be observed when the ratio *a*_w_/*x*_eq_ is plotted against *a*_w_ (Blahovec and Yanniotis 2009). The GAB model was adapted to experimental water absorption data for orange prickly pear juice powder and the estimated parameters are shown in Table 7.

**TABLE 7:** Equation and statistical parameters of GAB model for moisture absorption isotherms of spray dried prickly pear powder.

Parameters	Carrier systems				
	MD	WPI 1	WPI 100	GA	Cas
*X*_m_ (%d.b.)	6.36	6.01	5.36	5.46	5.39
*C*	4.77	5.02	7.15	6.02	5.95
*K*	1.02	1.03	1.05	1.06	1.07
*R* ^2^	0.999	0.998	0.999	0.998	0.999
*E*	2.69	4.17	2.27	2.92	2.50

The *x*_m_ values obtained for the various tests ranged from 5.36 to 6.36 g/100 g d.b. with the highest value corresponding to the plain maltodextrin system and the lowest to the WPI100 carrier system (5.36 g/100 g d.b.). Similar GAB parameter values were reported by Carmo et al. (2018) for spray dried beetroot juice with the same ratio of juice solids:carriers (1:2) and same ratio of MD:WPI (1:1), although it has to be noted that filtered beetroot juice has been used, in which sucrose is the main sugar, while prickly pear juice contains mainly glucose and fructose.

**FIGURE 3:**
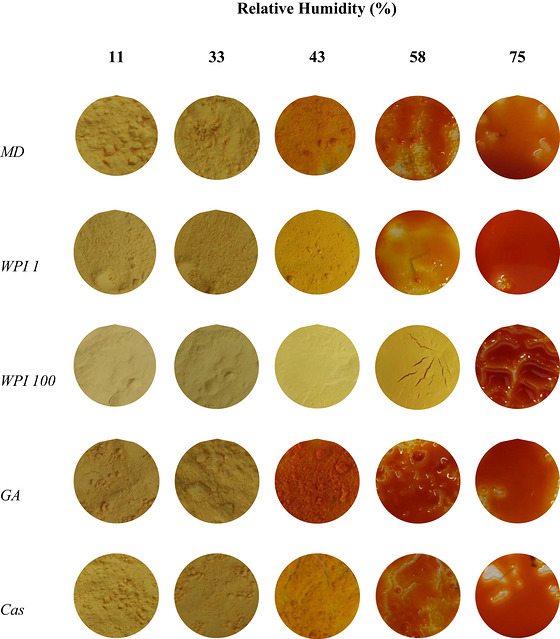
Prickly pear juice powders exposed to various water activities at 25°C until constant weight.

The effect of the carrier systems on the structural stability of the particles was significant. As shown in Figure 3, at 43% relative humidity a state transition started to take place in all systems except for the WPI100, which showed no signs of caking. Caking for WPI100 is only starting to appear in 58% relative humidity, where all the other systems exhibited a visible change in physical stability, probably due to the transition to the rubbery state. This finding is really important for the stability of the final prickly pear powder as it leads to extension of its self‐life. Prickly pear juice solids mainly contain fructose and glucose, which both have very low glass transition temperature (*T*_g_). The *T*_g_ further decreases as the powder absorbs water, which is a plasticizer with very low *T*_g_ (Bhandari et al. 1997). The relation between water content and *T*_g_ has been documented (Kaderides and Goula 2017). Carmo et al. (2018) studied the stability of spray dried beetroot juice encapsulated with the same juice solids:carriers ratio (1:2) and observed good stability to relative humidity up to 58% both for maltodextrin and for the mix of maltodextrin with whey protein. However, beetroot juice contains mainly sucrose, which has a higher *T*_g_.

## 4 Conclusions

Prickly pear juice was spray dried using different carrier systems. Increased whey protein isolate concentration led to better powder yield, although even a minimal amount of whey protein enhanced powder yield. Increased whey protein concentration also improved handling properties of the powder, as it hindered powder caking in higher relative humidity, compared to the other systems. Relative humidity conditions also play a very important role in the degradation of prickly pear betalains, while the partial substitution of maltodextrin by other carriers did not appear to play a major role in the preservation of betalains during storage. Production of prickly pear powder with spray drying is feasible and should take into consideration all these aspects to select the most adequate and cost‐effective carrier system according to the technical requirements of the final product. It is worth noting that a pilot‐plant validation of the results found would be of benefit of this research.

## Author Contributions

**Panagiotis Chaloulos**: investigation, writing – original draft, data curation. **Athina Mourouti**: investigation, methodology. **Ioanna Mandala**: conceptualization, funding acquisition, writing – review and editing, resources, supervision.

## Conflicts of Interest

The authors declare no conflicts of interest.

## Supporting information




**Supporting Information**: jfds71221‐sup‐0001‐FigureS1.docx
