# Supplementary figures and images for: Spray Drying of Prickly Pear (Opuntia ficus‐indica [L.] Mill) Juice: Effects of Different Carriers on Yield and Powder Properties
Source: J Food Sci. 2026 Jun 26;91(7):e71221. doi: 10.1111/1750-3841.71221 (PMC13306922; doi:10.1111/1750-3841.71221)

**Supplementary information:**


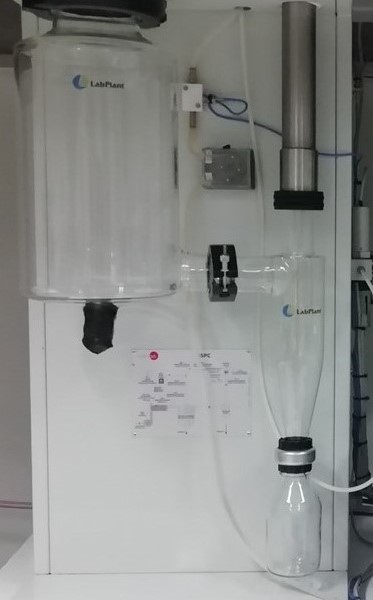


Figure S1: Spray drier unit (SSPC, Edibon,Spain)

Supplement: Supplementary file 1 — Supporting Information: jfds71221‐sup‐0001‐FigureS1.docx [file JFDS-91-0-s001.docx]
